# Addressing Health Inequities in the United States: A Case Report on Autism Spectrum Disorder (ASD) and Social Determinants of Health

**DOI:** 10.7759/cureus.42539

**Published:** 2023-07-27

**Authors:** Kristen E Fox, Adriana S Maribona, Juliana Quintero, Carlos Lange, Katherine Semidey

**Affiliations:** 1 Pediatrics, Florida International University, Herbert Wertheim College of Medicine, Miami, USA; 2 Florida Medical Student Research Journal (FMSRJ), Florida International University, Herbert Wertheim College of Medicine, Miami, USA

**Keywords:** social determinants of health (sdoh), global healthcare systems, case report, autism spectrum disorder, pediatrics

## Abstract

Autism spectrum disorder (ASD) is a complex neurodevelopmental disorder that presents with delays in developmental milestones, social impairment, and behavioral difficulties. Treatment relies upon an intensive individualized multidisciplinary medical and therapeutic approach. This case presents a child affected by ASD and other associated conditions, whose care was significantly limited by the effects of social determinants of health, including lack of transportation, housing instability, low income, and most importantly, lack of health insurance. Without universal health coverage, the US healthcare system requires that patients have insurance or pay out-of-pocket to access medical care. It is vital that healthcare providers are able to recognize and address these barriers in order to help pediatric patients and their families navigate a difficult and often inequitable healthcare system. It is also crucial to emphasize the importance of healthcare providers, policymakers, and advocacy organizations to work together to address the systemic barriers that limit access to quality care for children with ASD.

## Introduction

Autism spectrum disorder (ASD) is characterized by developmental delays in communication, socialization, and behaviors that can present in varying degrees based on the individual [1]. Some common comorbidities associated with ASD include attention-deficit/hyperactivity disorder (ADHD), anxiety disorders, depression, epilepsy, sleep disorders, and gastrointestinal and eating disorders [2-3]. These delays and comorbidities can significantly impact the quality of life and functioning of children with ASD, making early intervention and treatment essential to improving health outcomes for these individuals. Multidisciplinary interventions are recommended for children with ASD, as no one treatment has been deemed superior for improving issues in communication, behavior, and socialization [1]. Some of the most commonly used therapies include applied behavioral analysis (ABA) therapy, speech therapy (ST), occupational therapy (OT), physical therapy (PT), and social skills training [4]. The challenges and demands of caring for a child with ASD are amplified for those facing the repercussions of several social determinants of health, namely lack of health insurance and low socioeconomic status. Without insurance or the ability to pay out-of-pocket, these medically underserved families face far greater challenges accessing basic healthcare and ASD resources [5-6]. To add to their financial burden, mothers of children with ASD can suffer up to 75% loss of productivity, affecting household income dramatically [7]. These compounding factors often result in inadequate care for pediatric ASD patients, and the lack of interventions can trigger severe behavioral issues, which in turn worsen parental stress levels, feeding into this disruptive cycle [5].

This case report illustrates the importance of recognizing these barriers and the role that healthcare providers can play in helping children with ASD and their families navigate an extraordinarily complex and inequitable healthcare system.

## Case presentation

A five-year-old Honduran male with a history of moderate to severe ASD (impairments and behaviors require substantial support) and avoidant/restrictive food intake disorder (ARFID) presented to UHI Community Care Clinic, a free healthcare clinic in South Florida for the uninsured and low-income, to establish care. During the first encounter, the patient’s mother expressed concerns about her son’s diagnosis, not receiving sufficient therapies, and the impact that numerous barriers have had on his care. 

The patient presented to our clinic with severely delayed speech and unmet social and cognitive milestones. Per his mother, he is non-verbal and unable to understand verbal commands. He has also been exhibiting behavioral difficulties, becoming increasingly aggressive with multiple episodes of agitation per day. His mother notes that he often has difficulties sleeping, averaging only three to four hours nightly, and his aggression is worse on days after poor sleep. In the past, she managed his sleep difficulties with melatonin but claims that this is no longer working. Another major struggle for mom is his severe food restriction. He rejects all foods except for bread and water, putting him at serious risk of developing nutritional deficiencies. Despite these developmental delays and behavioral issues, the patient only has minor fine motor delays, such as the inability to write, trace, or color, but has no major gross motor delays. He is also occasionally affectionate and is able to perform tasks such as dressing and feeding himself. 

The patient was born in Honduras and came to the United States three years ago with his single mother and 16-year-old sister, who has mild ASD (impairments and behaviors require minimal support). Given his family’s current immigration status, the patient is not eligible for Medicaid. Lack of health insurance has prevented the patient from receiving primary care, specialist care, and necessary therapies such as ABA therapy, ST, OT, PT, and parent-child interaction therapy (PCIT). Language barriers and lack of transportation and primary care have made it difficult for his mother to identify and access local resources to obtain the level of care our patient requires. 

The patient had been connected to a local advocacy group for those with disabilities who paid for short-term ABA therapy through a grant. Per mom, these sessions improved his behaviors during the three months he received therapy. In September 2022, he underwent a psychoeducational evaluation, and his individualized education plan (IEP) was developed for school. Although his psychoeducational evaluation described his need for several therapies and services, his IEP only specified that he receive one hour of ST weekly with a speech and language therapist within the school. These therapy sessions are carried out in groups and are not individualized to address our patient’s specific needs. His mother has been unsure of how to advocate for him to receive more services in school and finds herself unable to communicate with his teacher due to language barriers. 

Social history 

The patient lives in an apartment with his mother and sister. The patient’s mother, the primary caregiver and only source of income for their family works as a kitchen assistant on the night shift. Their financial stability is precarious, given her low-paying job that provides her with a monthly income of only $740. She frequently worries about affording rent and relies heavily on food donations. Aside from his mother and sister, the patient is also often cared for by his maternal aunt who lives nearby with her husband and works cleaning houses. She will frequently care for the patient while his mom is working at night. Transportation has also been a serious barrier to the patient’s care, as the family does not have a car and depends on public transportation, which can often be unreliable and typically requires multiple transfers to reach their destination. Most importantly, however, the patient would have episodes of aggression and agitation on the bus, making it burdensome for his mother to travel long distances for his appointments at our free clinic. These same episodes of aggression and agitation have contributed to behavioral problems at school and at home while his mom is at work. These behavioral issues, in addition to the patient’s profound speech delays and limited vocabulary, have led to consequential learning and communication difficulties. Lastly, his mother endorses feeling extremely overwhelmed and stressed, raising grave concerns regarding her own mental health and well-being.

In this case, the providers carefully addressed each barrier to begin unloading the mother’s stress and to start accessing the ASD care the patient desperately required. The first steps were to address the patient’s more disruptive behavioral issues and poor sleep. Although he had received a financial assistance card from the local safety net hospital, the wait time for new patient evaluations in the Neurology clinic was six months. Therefore, at the second follow-up visit at the clinic, the decision was made to initiate low-dose Clonidine to target his aggression, sleep disturbance, and attention at school until he can be seen by a Neurologist. Per his mother, this has made a dramatic impact on his ability to sleep soundly at night, has reduced the frequency of aggressive outbursts, and has improved his behavior at school. She describes he is still a picky eater; however, since starting Clonidine, she has started to incorporate beans into his diet with some success and has been able to get him to occasionally drink Pediasure. The team was also successful in helping the family to gain special transportation service (STS) access, minimizing the emotional and financial burden of using public transportation. 

Moreover, his mother has been connected with resources to improve his quality of care. Through a local advocacy group, they have helped with pushing to receive additional services in school. She was also placed in contact with two local programs that provide limited but free speech and physical therapy to underprivileged children. Through one of these organizations, he has already received an initial speech evaluation and is awaiting initiation of speech therapy, one hour per week. However, we have been unable to access ABA therapy without insurance or the ability to pay out-of-pocket. 

Addressing another pressing concern, his mother’s health has been a priority to the providers at the clinic, working to ensure she gets the care she needs to be mentally and physically capable of caring for her children. The team worked to arrange her primary and psychological care within the free clinic to help her address her stress, anxiety, and depression.

## Discussion

This case report highlights the impact of social determinants of health on patients with ASD and the importance of addressing these factors to help patients obtain more access to the services they desperately need. The patient’s family struggles with multiple social determinants of health, including transportation, housing, child care, food insecurity, financial instability, and lack of health insurance. These obstacles have led to his inadequate access to treatment therapies and specialist care. This case made clear how healthcare providers can play a vital role in helping families navigate these challenges and access the care they need. 

With the increasing prevalence of ASD in the United States, there has been a shift in focus toward early interventions to reduce ASD costs, as it is estimated to cost the country $589 billion per year by 2030 [7]. Global healthcare systems differ widely in how nations organize and finance their medical systems. Here in the United States, we rely almost exclusively on health insurance to access affordable medical care, whether through for-profit insurers, non-profit insurers, or government-funded programs like Medicaid and Medicare. This leaves uninsured patients paying high prices out-of-pocket for medical services. Moreover, public schools are also responsible for bearing some of the burden of specialized care and education of special needs children like those with ASD. Structured and individualized educational plans can cost public schools more than $12,000 yearly per child with ASD [1, 7]. 

**Figure 1:**
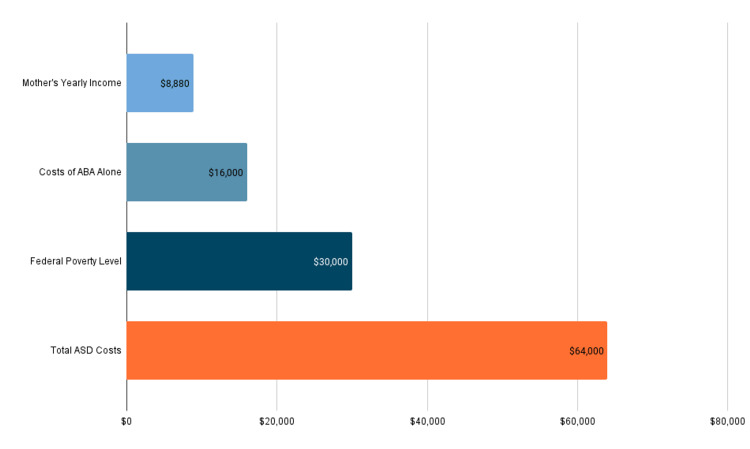
Comparing annual estimated ASD costs to the US federal poverty level. Comparing annual estimated ASD costs to the federal poverty level. The graph illustrates the financial burden faced by families of children with ASD, particularly those living in or below poverty level. Estimated total annual costs of caring for a child with ASD (total ASD costs), estimated annual costs of ABA therapy (costs of ABA alone), and the federal poverty level for a family of four in the United States in 2023 are shown [5, 7-8]. ASD, autism spectrum disorder

For children with ASD, recommended therapies for ABA therapy alone can reach over $18,000 per year for children under six years old [5]. With the mother’s annual income of $8,880, less than a third of the federal poverty level (FPL) for a family of four ($30,000), it was financially impossible for her to afford to pay out-of-pocket (Figure 1) [7-8]. More broadly, it is estimated that annual ASD care can amount to up to $64,000 per child, depending on the severity of the disorder, a cost our patient’s mother would never be able to bear alone. Figure 1 highlights the significant strain that ASD places on low-income families like theirs and underscores the need for increased support and resources for families caring for children with ASD. For low-income and uninsured patients with diagnoses that require individualized, complex, multifaceted care, the US healthcare system often falls painfully short of meeting their medical needs. These patients are left to rely upon nonprofit organizations and public school systems to fill the gaps.

For our patient, by utilizing local non-profit free therapy resources, we were successfully able to obtain 1 h of weekly ST, but still far below what he requires. Speech and language therapies aim to improve communication skills, while occupational therapy specifically addresses sensory and motor skills deficits. ABA therapy, however, takes a more holistic approach to complex patients with ASD and focuses on positive reinforcement to increase desirable behaviors specifically tailored to the patient’s needs. Studies have shown that at least 15 h weekly is recommended for ABA therapy to have an effective impact [5]. As the evidence-based best practice treatment for ASD, ABA therapy remains the therapy our patient most desperately needs to make positive improvements in his intellectual, language, and social functioning. Free resources for ABA therapy in our local area sadly do not exist. In more severe cases like our patient’s, pharmacological interventions can be integrated into care to target behaviors that impede benefits from other applied therapies [1]. In this case, Clonidine had a positive impact on his behaviors and may allow his therapy to be more effective. All of these elements together are necessary for quality, comprehensive ASD care that for uninsured, low-income patients like ours, is typically far out of reach.

Additionally, his mother’s mental health was a unique and critical aspect in treating this patient, as several studies have shown that caretaker burnout is common among parents with children with ASD, which can inevitably impact ASD outcomes [5]. The financial demands and psychological challenges of having a child with ASD significantly impact the mental and physical health of the parent or caretaker, increasing their risk for depression, stress, social isolation, and caretaker burnout [5, 7]. These effects are more prominent in single parents of children with ASD. The constant need to navigate complex healthcare and school systems, as well as manage challenging behaviors in their child, can exacerbate stress levels for parents of children with ASD [9]. Maintaining access to regular psychiatrist care for mom has been an incredibly important element of this child’s plan of care for these reasons. 

This case illustrates the importance of a healthcare provider's ability to recognize and address social determinants of health affecting their patients, as it is crucial in managing patients with ASD and associated conditions. Providers can play an essential role in helping families access necessary care by working with them to identify local resources and teaching them how to navigate a complicated, and oftentimes inaccessible healthcare system. In addressing these challenges, providers can improve health outcomes and quality of life of children with ASD, despite social or economic barriers. 

## Conclusions

Autism spectrum disorder is a neurodevelopmental disorder that affects social communication and behavior. It often presents with other conditions, such as sleep disturbances and ARFID. These complex disorders can be difficult to manage, particularly when complicated by social determinants of health. This was a case of a five-year-old male who presented for a well-child visit and evaluation of his ASD and ARFID, who was burdened with several social determinants of health, which gravely impaired his access to care. This article adds to the current literature by addressing specific social determinants of health that can affect children with ASD, emphasizing the critical role that providers play in helping these patients and families navigate a complex healthcare system. We focus here on global healthcare systems from the perspective of the United States, to highlight where this country falls short when it comes to providing uninsured children with special needs the care they deserve. This patient will continue to be evaluated at our free clinic for his ASD and address any nutritional and behavioral issues. However, comprehensive and complete care will unfortunately be out of reach for him until he can obtain health insurance. For now, support from non-profit organizations and other community-based resources will be essential in addressing this patient’s needs and improving the quality of life for both him and his family. 
